# An Innovative Traction-free Drugless Approach to Reduce Anterior Shoulder Dislocation: Axillary Massage Technique

**DOI:** 10.1016/j.acepjo.2024.100017

**Published:** 2025-01-10

**Authors:** Ayşegül Islek Yuksel, Abdullah Ortadeveci, Nalan Kozaci, Osman Polat, Serkan Yuksel

**Affiliations:** 1 Department of Emergency Medicine, Kahta State Hospital, Adiyaman, Türkiye; 2 Department of Anatomy, Faculty of Medicine, Eskisehir Osmangazi University, Eskisehir, Türkiye; 3 Department of Emergency Medicine, Alanya Alaaddin Keykubat University, Faculty of Medicine, Antalya, Türkiye; 4 Department of Orthopedics and Traumatology, Adıyaman Training and Research Hospital, Adıyaman, Türkiye; 5 Department of Radiology, Medical Park Mersin Hospital, Mersin, Türkiye

**Keywords:** shoulder dislocation, shoulder reduction, reduction technique, emergency

## Abstract

Although shoulder dislocation is one of the most common cases encountered in emergency departments, there is still no consensus on the method of reduction. Here, we reported 9 cases in which we used a new technique that did not require any traction, medication, or patient effort. This technique can give results in 20 seconds in some cases and provides high patient satisfaction. Due to its easy applicability and high success rate, we think that the technique will make a significant contribution to the literature and clinical practice.

## 1 Introduction

Shoulder dislocations commonly occur in young males and as a result of trauma. In clinical practice, anterior dislocations are most frequently observed. Patients often present to the emergency department with shoulder deformities, severe pain, and impaired mobility. Many methods are currently used for the reduction of anterior shoulder dislocation. In some cases, more than one technique can be used for reduction.^1^, ^2^, ^3^, ^4^ Because there is no single technique that can offer a solution in every case, it is a serious advantage for the practitioner to be familiar with different techniques.

In 2003, Cunningham described a new technique for anterior dislocation reduction that is fast, painless, and drug-free. This technique is based on the hypothesis that muscle spasm is the main force that moves the humeral head away from the glenoid cavity in anterior dislocations. Therefore, the reduction procedure is focused on the relaxation of the biceps brachii muscle by massage.^5^^,^^6^ Several studies have shown that the Cunningham technique has been successfully practiced and is still one of the most used shoulder reduction techniques in emergency departments.^7^^,^^8^

This presentation explains the “Islek Axillary Massage Technique,” which has been successfully utilized in 7 cases of anterior shoulder dislocation to date (see Supplementary Video). The Islek technique has features both similar to and different from those of the Cunningham. Although it is similar to the Cunningham technique in terms of providing reduction drug-free, painless, and with only massage, it has some differences in terms of the region massaged and not requiring patient effort.

**SUPPLEMENTARY Video:**
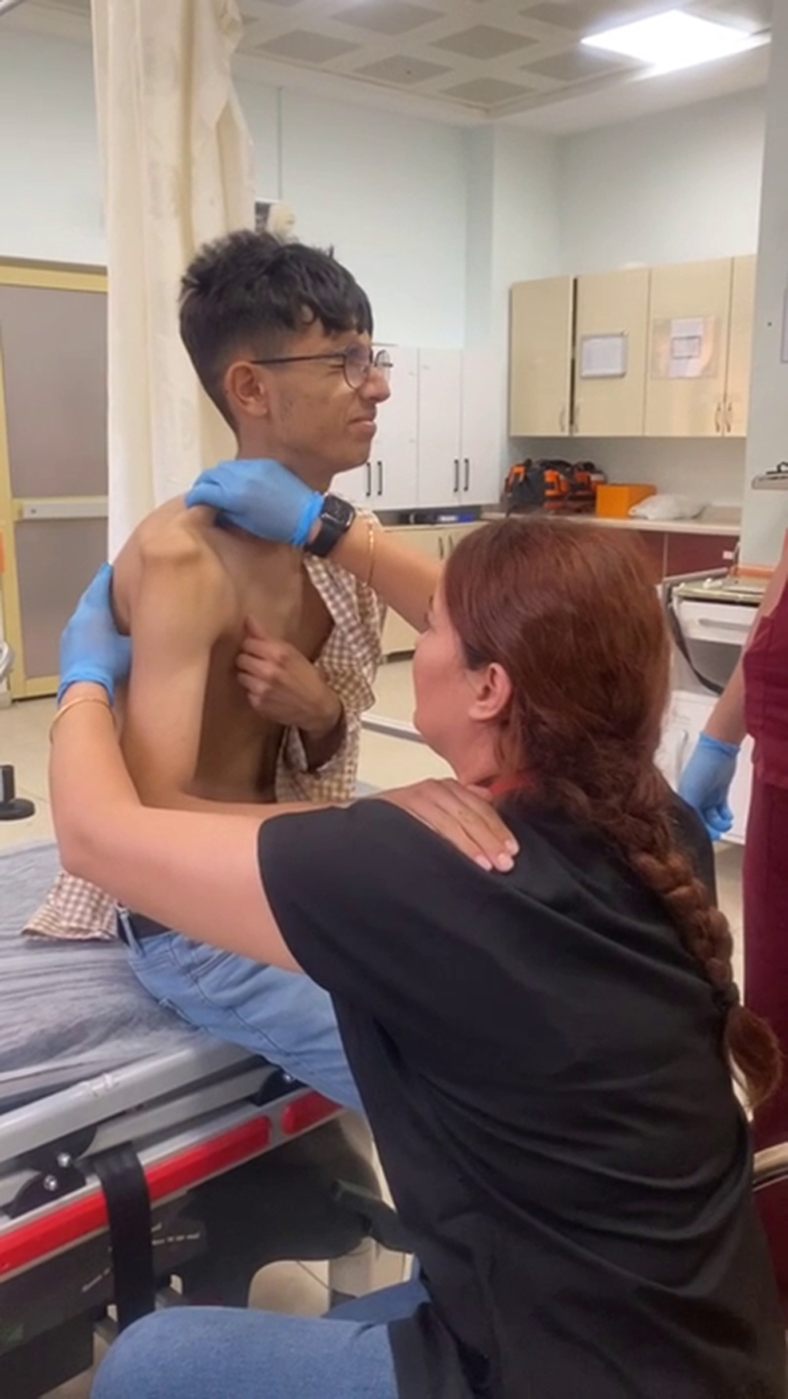
Demonstration of the Islek axillary massage technique.

## 2 Islek Axillary Massage Technique Application Steps

The ideal patient for this technique is a patient with anterior shoulder dislocation who is conscious, able to communicate, calm, and willing to participate in the procedure. It should be explained to the patient that she/he will only be massaged and that no extra pain will be caused. The practitioner should explain to the patient that the muscles will gradually relax with the massage. During the execution of the technique, no force is applied to any part of the body, such as pushing/pulling, and the patient is not asked to make any movement. In the meantime, the practitioner can start a mini-general conversation. This will prevent the patient from focusing on the reduction procedure and prevent voluntary/unintentional contractions in the muscles of the region.

1. The patient is seated opposite the examiner, with his/her back straight. The practitioner should sit in a lower position. The practitioner's head should be in line with the patient's shoulder. If the patient has right shoulder dislocation, place the forearm and hand on the practitioner's left shoulder in the position that causes the least pain. The practitioner should start the massage with the left hand and left thumb to massage the posterior axillary fold effectively. If the patient's left shoulder is dislocated, these steps are performed symmetrically.
2. The patient's dislocated shoulder should be held in 60 to 90º of flexion and internal rotation, ie, in its original position. The patient's forearm is bent 90 to 120º. The patient's wrist should be on the practitioner's shoulder in pronation and 10 to 30º extension. In this position, the patient's pain is relieved as the weight of the dislocated shoulder is supported. Any pulling movement should not be applied as this will cause pain and result in spasms.
3. The practitioner places one hand on the patient's shoulder and the other on the axilla.
4. First, the practitioner starts massaging the posterior axillary fold (latissimus dorsi and teres major muscles) in the deepest part of the axilla with the thumb.
5. With the other hand, the practitioner gently massages the upper part of the patient's trapezius muscle.
6. The scapula is placed in a retroverted position by means of maneuvers in the axilla. During the massage, the humeral head is placed into the glenoid cavity spontaneously with the relaxation of the latissimus dorsi and teres major muscles.
7. It is not recommended that the practitioner has long fingernails as this prevents effective massage and traumatizes the patient's armpit.

The technique is designed to be used without medication. In cases where the technique is performed, after proper massage, reduction can take place at any time between 20 seconds and 5 minutes. In shoulders that do not reduce with this technique, other techniques appropriate to the patient should be used.

## 3 Anatomical Basis of the Technique

The Islek technique works based on 2 basic anatomical principles. The first is to eliminate the adduction forces that cause the humeral head to remain medial to the glenoid cavity. The second is to remove the force that suspends the scapula and moves the articular surfaces away from each other. After anterior dislocation of the shoulder, the humeral head is often positioned subcoracoid or subglenoid. The contracted latissimus dorsi and teres major muscles, powerful adductors of the arm, are one of the main forces keeping the humeral head adducted.^9^ These 2 muscles form the posterior axillary fold and can be palpated on the posterior wall of the axilla. Relaxation of the muscles with massage results in lengthening them and thus decreases the adductor and internal rotator effect on the proximal humerus. Concurrently, the proximal humerus, which has shifted inward and anteriorly, moves outward and posteriorly under the influence of the contracted infraspinatus and teres minor muscles. This force causes the humeral head to cross the glenoid rim and settle in the glenoid cavity. On the other hand, the descending part of the trapezius muscle (superior fibers) is massaged. When the fibers of the trapezius contract, they pull the scapula superomedially (suspends).^10^ In a dislocated shoulder, this muscle suspends the scapula, causing it to keep away from the humeral head. Massage of the trapezius muscle relaxes the fibers, allowing the scapula to descend and thus be in the same plane as the humeral head.

## 4 Discussion

When choosing the technique for shoulder reduction in emergency settings, many factors such as reduction time, pain intensity during reduction, need for medication, occurrence of iatrogenic complications during reduction (neurovascular injury, fracture), and success rate of reduction should be evaluated.^1^

The Cunningham technique is based on the elimination of static obstruction with the patient moving the shoulders backward and upwards. In dynamic obstruction caused by biceps brachii muscle spasm, the muscle is relaxed with massage at the level of the mid humerus.^8^ In contrast, in the Islek technique, the patient is passive, and no movement is requested from the patient. The practitioner massages the posterior axillary crease, not the biceps brachii muscle. Within this fold are the latissimus dorsi and teres major muscles, which are the most effective forces keeping the humeral head in adduction. Relaxation of these muscles by massage reduces the adductor and internal rotator effect on the proximal humerus. On the other hand, the humeral head, under the force of the infraspinatus and teres minor muscles, moves outward and posteriorly. The practitioner additionally massages the upper part of the trapezius muscle on the patient's shoulder with the other hand. Relaxation of this muscle releases the suspended scapula.

In addition, the Islek technique does not require the patient's hand to touch the practitioner's chest, which is especially advantageous for female practitioners.

## 5 Conclusion

The Islek technique is a shoulder reduction method focused on muscle relaxation. No traction is applied in this technique. Therefore, it does not require force. The pain level is low because no force is applied during the procedure. Sedative and analgesic agents are not required. It can be performed by a single person without the need for an assistant. The patient passively participates in the procedure. Reduction time is quite short when the practitioner and patient are in harmony. However, the technique has been applied in a limited number of patients by specific practitioners. Further studies are needed to verify the efficacy of the technique and to investigate its pros and cons in more detail.

## Funding and Support

This research did not receive any specific grant from funding agencies in the public, commercial, or not-for-profit sectors.

## Conflict of Interest

All authors have affirmed they have no conflicts of interest to declare.
